# Analysis and Modeling of Rat Gait Biomechanical Deficits in Response to Volumetric Muscle Loss Injury

**DOI:** 10.3389/fbioe.2019.00146

**Published:** 2019-06-19

**Authors:** Jack A. Dienes, Xiao Hu, Kevin D. Janson, Conrad Slater, Emily A. Dooley, George J. Christ, Shawn D. Russell

**Affiliations:** ^1^Biomedical Engineering Department, University of Virginia, Charlottesville, VA, United States; ^2^Mechanical and Aerospace Engineering Department, University of Virginia, Charlottesville, VA, United States; ^3^Department of Orthopaedic Surgery, University of Virginia, Charlottesville, VA, United States

**Keywords:** muscle, kinematics, gait analysis, volumetric muscle loss, biomechanics, motion capture

## Abstract

There is currently a substantial volume of research underway to develop more effective approaches for the regeneration of functional muscle tissue as treatment for volumetric muscle loss (VML) injury, but few studies have evaluated the relationship between injury and the biomechanics required for normal function. To address this knowledge gap, the goal of this study was to develop a novel method to quantify the changes in gait of rats with tibialis anterior (TA) VML injuries. This method should be sensitive enough to identify biomechanical and kinematic changes in response to injury as well as during recovery. Control rats and rats with surgically-created VML injuries were affixed with motion capture markers on the bony landmarks of the back and hindlimb and were recorded walking on a treadmill both prior to and post-surgery. Data collected from the motion capture system was exported for *post-hoc* analysis in OpenSim and Matlab. *In vivo* force testing indicated that the VML injury was associated with a significant deficit in force generation ability. Analysis of joint kinematics showed significant differences at all three post-surgical timepoints and gait cycle phase shifting, indicating augmented gait biomechanics in response to VML injury. In conclusion, this method identifies and quantifies key differences in the gait biomechanics and joint kinematics of rats with VML injuries and allows for analysis of the response to injury and recovery. The comprehensive nature of this method opens the door for future studies into dynamics and musculoskeletal control of injured gait that can inform the development of regenerative technologies focused on the functional metrics that are most relevant to recovery from VML injury.

## Introduction

Volumetric muscle loss (VML) is characterized as an injury that exceeds the intrinsic regenerative capacity of skeletal muscle and results in irrecoverable tissue loss and permanent functional impairment (Grogan and Hsu, 2011). VML can be caused by a wide variety of conditions, including disease, surgical procedures, congenital anomalies, and traumatic injury suffered by both civilians and military personnel. Significant preclinical research into therapies for regeneration of the lost muscle volume is underway, with strategies including various combinations of scaffolds, hydrogels, and exercise regimens (Wu et al., 2012; Corona et al., 2013, 2015, 2017; Cittadella Vigodarzere and Mantero, 2014; Christ et al., 2015; Grasman et al., 2015; Dziki et al., 2016; Passipieri and Christ, 2016; Ma et al., 2017; Passipieri et al., 2017, 2019). These therapies have seen some success in terms of volume reconstitution, tissue remodeling, and recovery of force generation in the injured muscle. To date, improved force generation ability has been considered the most important and physiologically relevant index of muscle repair/regeneration following implantation of regenerative therapeutics. However, complete restoration of contractile function following treatment of VML injury has yet to be achieved, and human studies have demonstrated that increased strength (force generation capacity) does not necessarily result in increased movement function (Topp et al., 1993; Buchner et al., 1997; Damiano and Abel, 1998; Damiano et al., 2010, 2013). Moreover, the relationship between VML-related force deficits and gait biomechanics is not established in any biologically-relevant preclinical animal model that we are aware of. The goal of this study was to establish a robust and reproducible method to quantify the biomechanical changes in rat gait following a surgically-created VML injury to the tibialis anterior (TA) muscle.

Gait analysis of rat walking was chosen as a study parameter because it is the minimal reproducible functional level that could be reliably evaluated in all animals. Using a combination of motion capture and advanced musculoskeletal modeling techniques, it is possible to accurately measure the effects of these severe muscle injuries on the 3D joint kinematics of rats and use this information to develop conclusions about the response to injury. Motion analysis has been used on humans for years to characterize musculoskeletal pathologies by quantifying function in terms of joint kinematics (An and Chao, 1984; Astephen et al., 2008) and we sought to apply that same methodology to a rat model of VML.

The biological relevance of the TA VML injury has been previously shown (Sicari et al., 2012; Wu et al., 2012; Aurora et al., 2014) and the rat model of VML injury is advantageous because methods have been established for assessment of force generation ability (Wu et al., 2012; Aurora et al., 2014; Corona et al., 2015; Mintz et al., 2016). In addition, rats have been successfully utilized as models to predict physiological changes in humans for multiple pathologies including cardiovascular disease (Iannaccone and Jacob, 2009), osteoarthritis (Clarke et al., 1997; Ängeby Möller et al., 2012), spinal cord injury (Canu and Garnier, 2009; Perrot et al., 2009; Johnson et al., 2012; Datto et al., 2016), and Parkinson's (Deumens et al., 2002; Duty and Jenner, 2011). The osteoarthritic model of rats has been utilized to analyze changes in gait kinematics (Roemhildt et al., 2010) and spatiotemporal parameters (Ängeby Möller et al., 2012), but the authors did not rigorously evaluate underlying patterns of compensation.

Similarly, rats with spinal cord injury have been subjected to gait analysis via various acquisition techniques. Some studies utilized CatWalk (Datto et al., 2016) to measure stride length, walking speed, and weight bearing but did not provide specific information on joint kinematics. Another study on rat cadavers utilized X-ray (Wehner et al., 2010) to track the joint angles during simulated walking, but this is not an accurate method of analyzing movement function. In order to truly understand what is occurring physiologically and biomechanically, it is necessary to have a reproducible and reliable collection method as well as a data set that comprehensively defines the motion at all three joints throughout the entire gait cycle. More recently, advanced motion capture techniques (Vicon) typically reserved for humans have started being applied to rat models (Johnson et al., 2008; Webb et al., 2011; Eftaxiopoulou et al., 2014; Karakostas et al., 2014). However, 3D gait evaluation has not been performed on rats with VML injury to assess the extent of injury and recovery on movement function, as the focus of current VML treatments is primarily on recovery of force production ability and volume reconstitution (Corona et al., 2013; Aurora et al., 2014; Passipieri et al., 2017).

The present study examined the changes in gait kinematics of rats with VML injury walking on a treadmill. Using 3D motion capture, we developed a methodology to quantify visually observed variations in gait biomechanics and reveal the underlying effect of VML injury on joint kinematics and adaptation or recovery over time. We report here, for the first time, quantifiable kinematic gait alterations in all three axes associated with VML-induced force deficits to the TA muscle in an established rat model. These findings have major implications for evaluating and quantifying the efficacy of both regenerative and rehabilitative therapeutics for the treatment of extremity VML injuries. More generalized applications to the improved understanding and treatment of other forms of extremity injuries or disorders are also envisioned.

## Materials and Methods

### Experimental Outline

Eight 12-week old female Lewis rats were divided into two groups: four animals given 20% by mass VML injuries to their right TA with no repair performed (NR) and four healthy (no sham surgery) age match controls (Control). The gait biomechanics of both groups were analyzed using a combination of Vicon Nexus motion capture software and OpenSim modeling. Baseline motion capture and force testing occurred 1 week prior to surgery, and these measurements were repeated at 2, 4, and 8 weeks post-surgery to track the effect of the injury and subsequent recovery. The resulting kinematic and functional data from each timepoint was compared to the data collected at baseline to determine any significant differences.

### Animal Care

This study was conducted in compliance with the Animal Welfare Act, the Implementing Animal Welfare Regulations, and in accordance with the principles of the Guide for the Care and Use of Laboratory Animals. The University of Virginia Animal Care and Use Committee approved all animal procedures. A total of 8 female Lewis rats (Charles River Laboratories) weighing 180.2 ± 6.75 g at 12 weeks of age were pair housed in a vivarium accredited by the American Association for the Accreditation of Laboratory Animal Care, and they were provided with food and water *ad libitum*. The average animal body weights between the groups did not significantly differ over the course of the study.

### Anesthesia and Analgesia

All surgical and mechanical testing procedures as well as motion capture marker placement were conducted under anesthesia with continuous inhalation of isoflurane (1.5–2.5%). The depth of anesthesia was monitored by the response of the animal to a slight toe pinch, where the lack of response was considered the surgical plane of anesthesia. Core temperature was maintained using a heated water perfusion system. Rats were administered slow release buprenorphine (0.1 mg/kg, subcutaneously) prior to surgery and quick release buprenorphine (0.1 mg/kg, subcutaneously) at 36 and 48 h post-surgery. Animal pain and distress were monitored daily by qualified members of the veterinary staff to determine the need for additional analgesia. No animal required additional analgesia after 48 h post-surgery.

### TA VML Surgery

**Figure 1:**
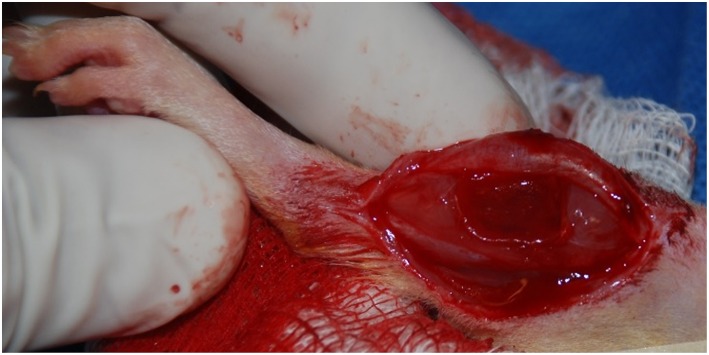
Representative surgical defect of ≈20% by mass volumetric muscle loss injury (1.0 × 0.7 × 0.2 cm).

The surgical procedure for the creation of a VML injury in the rat TA muscle is depicted in Figure 1. Using aseptic technique, a longitudinal incision was made on the lateral portion of the lower right leg. The skin was then cleared from the underlying fascia using blunt separation, and the fascia covering the anterior crural muscles was separated using blunt dissection. The proximal and distal tendons of the Extensor Hallicus Longus (EHL) and Extensor Digitorum Longus (EDL) muscles were then isolated and ablated. As previously described (Wu et al., 2012; Corona et al., 2013), the TA muscle corresponds to 0.17% of the gross body weight. The VML injury model was characterized by excision of roughly 20% of the TA muscle weight from the middle third of the muscle belly. The fascia was closed with 6-0 vicryl sutures and the skin was closed with 5-0 prolene using interrupted sutures. Skin glue was applied over the skin sutures to help prevent the incision from opening. No animals required additional surgical attention after the initial procedure.

### *In vivo* Force Testing

*In vivo* force testing was performed as previously described (Mintz et al., 2016). Briefly, at −1, 2, 4, and 8 weeks relative to the surgery date, rats were anesthetized and the right hind limb was aseptically prepared. The rat was placed in a supine position on a heated platform and the right knee was bent to a 90° angle. The leg was secured using a stabilizing rod and the right foot was taped to a footplate. The footplate was attached to the shaft of an Aurora Scientific 305C-LR-FP servomotor, which was controlled using a computer. Sterilized percutaneous needle electrodes were carefully inserted into the skin of the lower right leg for stimulation of the right common peroneal nerve. Electrical stimulus was provided using an Aurora Scientific stimulator with a constant current SIU (Model 701C). Needle electrode placement was optimized with a series of 1 Hz pulses resulting in twitch contraction (Mintz et al., 2016). Contraction of the anterior crural muscles leading to dorsiflexion of the foot was determined by measuring the maximal isometric tetanic torque over a range of stimulation frequencies sufficient to result in plateau of the torque response (10–150 Hz). Torque at baseline was normalized by the body weight of each animal. Torque at each post-surgical timepoint was normalized by the body weight of each animal on the day of collection, then was normalized to a percent of the baseline for that animal. The normalized torques at each post-surgical timepoint were averaged within the group for analysis. After force testing, the animals were allowed to recover on the heated platform and were then returned to the vivarium. For terminal timepoints, animals were euthanized via CO_2_ inhalation and cervical dislocation was performed as a secondary measure.

### Treadmill and Motion Capture

**Figure 2:**
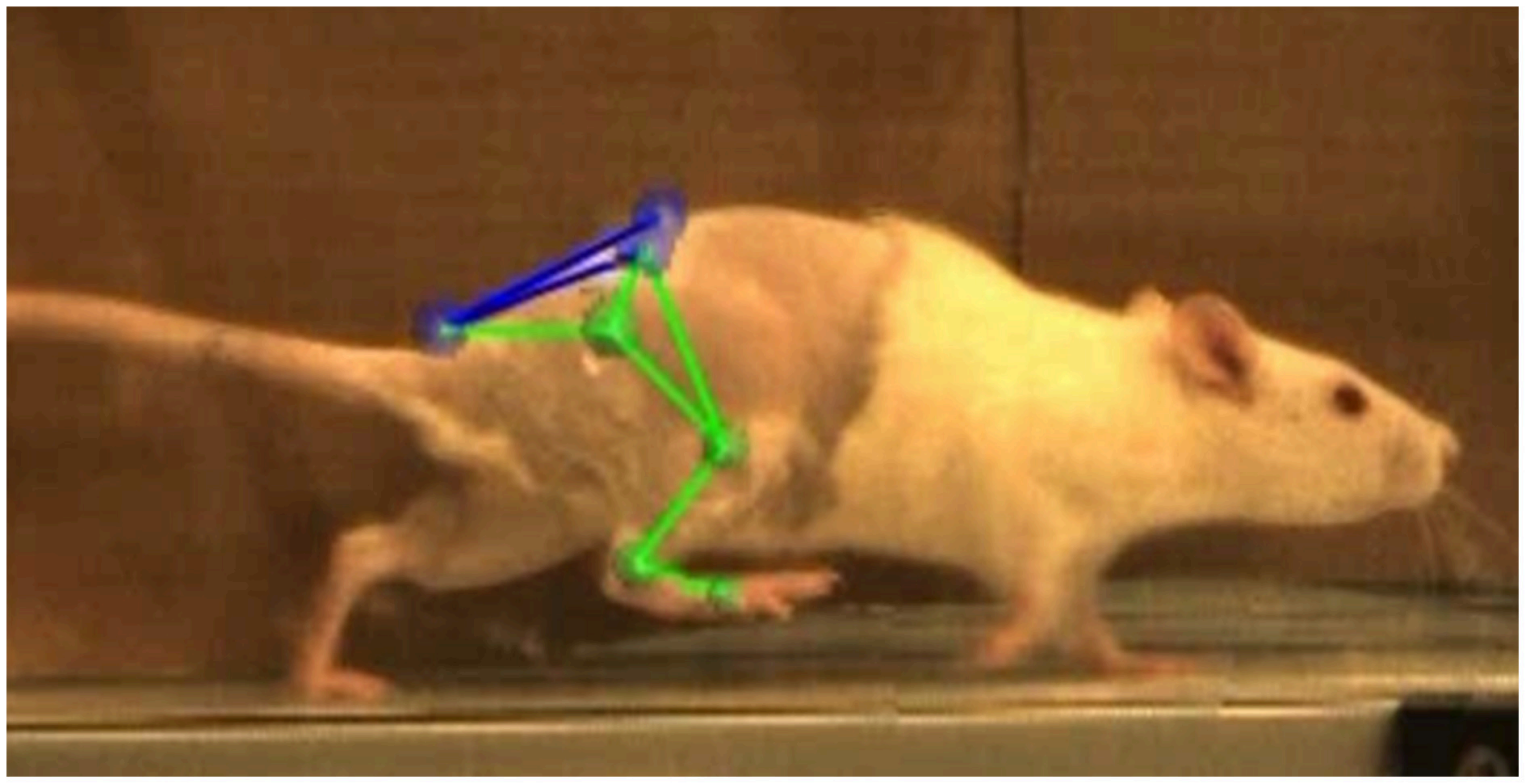
Vicon Nexus 2.8.1 3-D overlay of motion capture markers. Markers were placed on the L6 vertebra of the spine (SPINE), caudal-5 vertebra of the tail (TAIL), left and right anterior superior iliac crests (LASI, RASI), hip (HIP), lateral knee (KNEE), ankle (ANKLE), and 5th metatarsal on the toe (TOE).

In the week prior to the baseline motion capture session, rats were placed in the treadmill for two 20-min acclimation periods. They remained in their cages with no continued training for all post-surgical timepoints. At −1, 2, 4, and 8 weeks relative to the surgery date, rats were anesthetized and shaved to allow proper placement of the motion capture marker set illustrated in Figure 2. Reflective markers were placed on the bony landmarks of the left anterior superior iliac crest (LASI), right anterior superior iliac crest (RASI), spine (L6 vertebra), tail (5th caudal vertebra), hip, lateral knee, ankle, and distal end of the fifth metatarsal. Markers were always applied with the rats sedated and in the same body position in order to limit error due to skin movement and maximize repeatability in precise placement of markers on joint centers. Rats were allowed to recover from anesthesia on a heated mat before being placed in the treadmill. Kinematic data was collected using a Vicon 7-camera (T40) setup collecting at 120 Hz. Treadmill speed was set to 40 cm/s, a velocity safely in the reported range for walking speed in rats (Clarke, 1991; Pereira et al., 2006). The intensity of the shock at the rear of the treadmill was set to 0.1 mA to encourage the animals to remain on the belt and continue walking. Treadmill sessions lasted roughly 15 min per rat. After data collection the animals were returned to the vivarium.

### Gait Modeling and Statistical Analysis

**Figure 3:**
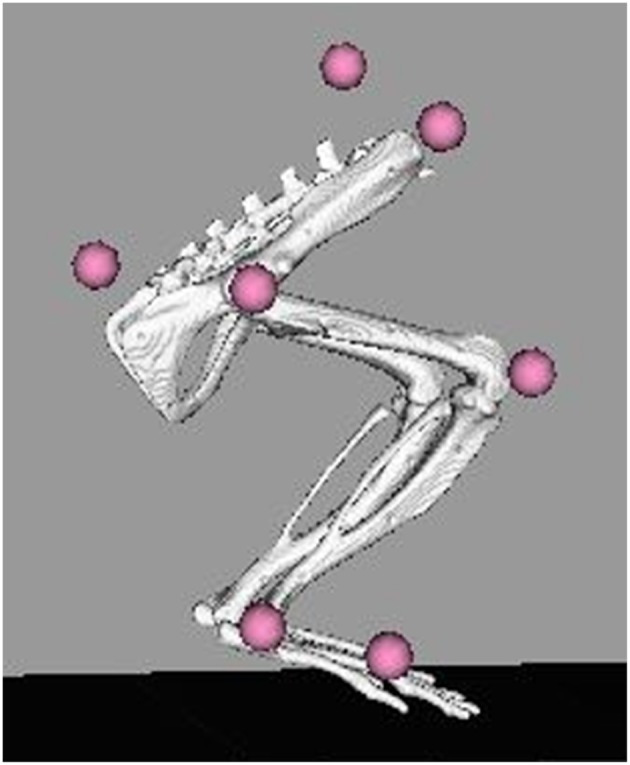
OpenSim rat hindlimb model with reconstructed marker locations. Marker locations shown are for the SPINE, TAIL, RASI, HIP, KNEE, ANKLE, and TOE. The model was scaled for each individual rat at each collection timepoint based on the locations of these markers on the animal.

Gait events and marker identification were completed in Nexus and converted to TRC format using a MATLAB script. Inverse kinematic modeling was performed in OpenSim using a modified version of an existing rat hindlimb model (Johnson et al., 2008) (Figure 3). This model consisted of four segments (pelvis, femur, tibia, foot) and had 13 degrees of freedom. The hip and ankle were modeled as ball joints, and the knee was modeled as a hinge joint. Resulting measures included sagittal joint angles of the hip, knee, and ankle as well as frontal and transverse angles of the hip. The model was modified to allow scaling to individual rats and facilitate calculation of joint angles through inverse kinematic simulations. As described in the previous section, the joints were palpated with the rat sedated and markers were placed directly over the joint centers of the hindlimb. This allowed limb segment lengths to be calculated from the motion capture data with the rat at a known position so the model segments could be scaled appropriately. The knee and ankle were limited to sagittal movement and the limbs segments were modeled as rigid bodies. Together, this facilitated full 3D analysis of kinematics while reducing the number of motion markers. Skin artifact is always an issue in motion capture and working with rats was no exception. We chose to analyze simple walking motion as it produced minimal skin to skeletal motion everywhere except for the knee, where this relative motion was still significant. To account for this, the weight/motion contribution of the knee marker was reduced in the sagittal plane, thereby reducing its error contribution to the modeled motion. Sagittal knee angles were calculated using the OpenSim rigid body model and driven by the hip/ankle markers, which tracked the limb motion with higher accuracy. This allowed us to account for the movement of skin over the knee joint in the sagittal plane without losing the motion data. Reported data corresponds to one gait cycle, heel strike to heel strike of the right leg. Heel strike and toe off events were identified through calibrated, synchronized high-speed video that was captured alongside marker data and 3-D overlain with the motion capture marker positions (see Figure 2). A minimum of three steps per rat at each timepoint were used for statistical analysis, with the exception of one NR rat at the 4-week timepoint where lack of willingness to walk on the treadmill resulted in only two usable trials.

Statistical analysis of the force testing data was performed using GraphPad Prism. A one-way ANOVA (α = 0.05) with multiple comparisons and Fisher's post-test was used to compare each of the post-surgery data sets to the baseline data and to each other. Analysis of the joint kinematic curves was performed using Statistical Parametric Mapping (SPM1) MATLAB code and *t*-tests. Spatiotemporal parameters were evaluated using paired *t*-tests (α = 0.05).

## Results

### Force Testing

**Figure 4:**
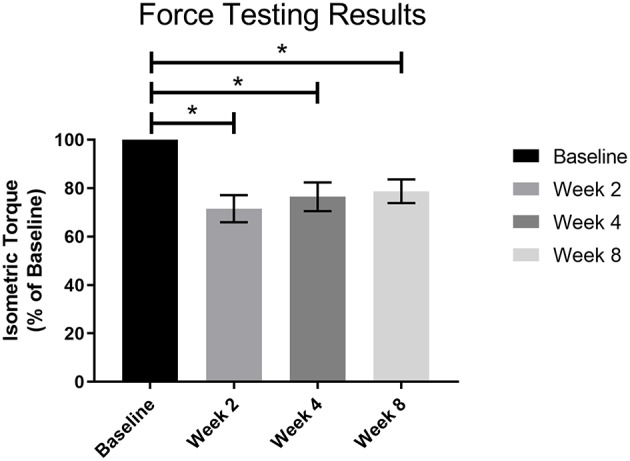
Force testing results for all VML animals (*n* = 4) prior to surgery and 2, 4, and 8 weeks post-surgery. There was a significant functional deficit at all post-surgical timepoints as compared to baseline (*p < 0.05), and no notable recovery as the post-surgical results were not significantly different from each other. Statistics were run as a one-way ANOVA with multiple comparisons and Fisher's LSD post-test. *N* = 4 for all timepoints.

Post-surgical force testing at 2, 4, and 8 weeks indicated a significant deficit in isometric tetanic dorsiflexion torque at all three timepoints relative to baseline in the NR group. The average baseline torque for the NR group was 113.90 ± 5.34 Nmm/kg. Average torque as percentage of baseline at Week 2 was 71.51 ± 5.59% (81.50 ± 8.16 Nmm/kg, *p* < 0.01), at Week 4 was 76.48 ± 5.90% (87.21 ± 9.53 Nmm/kg, *p* < 0.01), and at Week 8 was 78.74 ± 4.85% (89.70 ± 7.31 Nmm/kg, *p* < 0.01, Figure 4). The torques measured at 2, 4, and 8 weeks were not significantly different from each other (W2vW4, *p* = 0.48; W2vW8, *p* = 0.19; W4vW8, *p* = 0.90).

### Spatiotemporal Parameters

Average baseline spatiotemporal parameters are a composite of all rats, and a minimum of three steps per animal were averaged at the 8-week timepoint. Measurements of cadence (steps per minute, spm), step time (seconds), and swing percentage at Week 8 were compared to baseline. Over the course of the study, cadence significantly increased (172.07 ± 16.77 spm vs. 200.05 ± 30.44 spm, *p* < 0.05), stride time significantly decreased (0.352 ± 0.034 s vs. 0.314 ± 0.059 s, *p* < 0.05), and swing percentage significantly decreased (45.71 ± 3.23% vs. 41.82 ± 2.78%, *p* < 0.05).

### Joint Kinematics

**Figure 5:**
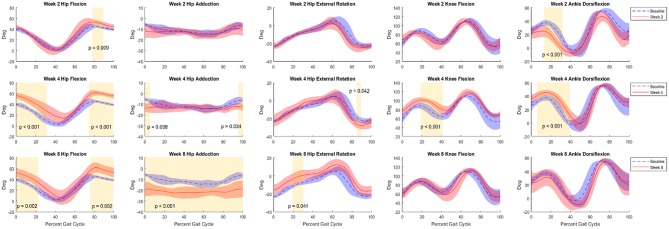
Kinematic curves for hip flexion, hip adduction, hip external rotation, knee flexion, and ankle flexion. Graphs shown compare baseline vs. 2 weeks (top row), 4 weeks (middle row), and 8 weeks (bottom row) post-surgery. Baseline data (Control+NR, *n* = 8) are shown in blue and post-surgical (NR, *n* = 4) data are shown in red. Regions of significance are shaded yellow with the respective *p*-value noted. All statistical analyses of kinematics were *t*-tests performed at an α-level of 0.05.

Average baseline kinematics are a composite of all rats (*n* = 8). Compared to baseline data, analysis of joint kinematics of the hip, knee, and ankle of the VML animals (*n* = 4) at −1, 2, 4, and 8 weeks showed significant differences across the board (Figure 5). Observed average ranges of motion for flexion of the hip, knee, and ankle joints were 39.24 ± 6.99, 52.88 ± 10.21, and 53.88 ± 10.07 degrees.

At Week 2, primary differences were seen in hip flexion in late stance (decrease, *p* < 0.001), early stance and heel strike hip adduction (decrease, *p* < 0.05), hip external rotation at toe-off (decrease, *p* < 0.05), mid-stance and mid-swing knee flexion (decrease, *p* < 0.001), and ankle flexion throughout stance and through swing (decrease, *p* < 0.001). By Week 4 the trajectories adapted such that primary differences were observed in both early stance and late swing for hip flexion (increase, *p* < 0.05 and *p* < 0.01) and adduction (decrease, *p* < 0.05 and *p* < 0.001), and ankle flexion at toe-off through mid-swing (decrease, *p* < 0.05). At the 8-week timepoint, the differences in hip flexion during late swing (increase, *p* < 0.01) were sustained. Hip adduction exhibited differences across the duration of the gait cycle (decrease, *p* < 0.001) and external rotation showed differences through mid-stance and mid to late swing (increase, *p* < 0.001) showed significant differences across the duration of the gait cycle. Knee flexion showed differences during early stance and late stance through toe-off (decrease, *p* < 0.01 and *p* < 0.001) and ankle flexion showed differences throughout stance and into mid-swing (decrease, *p* < 0.001). Additionally, a delayed phase shift was observed in all joint angles as the weeks progressed. By Week 8, the delay of hip flexion, hip adduction, hip external rotation, knee flexion, and ankle flexion was 3, 2, 3, 5, and 4%, respectively.

## Discussion/Conclusion

At the outset of this study, we sought to examine a more comprehensive approach to evaluating the effect of VML injury and recovery on rats by analyzing gait biomechanics rather than simply force generation ability. By collecting motion capture data on rats walking on a treadmill, we were able to delineate clear differences between the gait of healthy animals when compared to injured animals which was quantifiable via kinematic analysis (Figure 5). Healthy animals without sham surgery were included in this study and evaluated over the 8 weeks partially to ensure that there were no gait changes with age and growth. We found that there were no significant differences in mean or variance between their baseline and 8-week gait patterns. This, along with the demonstrated sensitivity to measure significant differences between healthy and VML injured rats, gives us confidence in the reproducibility of the methods employed in this study. With this in mind, we measured significant differences at all three major joints in the hindlimb and observed clear changes in adaptation in the gait patterns throughout the recovery period. It is possible that some of the kinematic changes could have been due to the surgical procedure, but in the human population there will always be a procedure performed to address the injury so we consider any noise from the surgery to be a part of the injury/recovery process.

As a standard of comparison, we referred to the baseline joint kinematics collected on each of the rats. The range of motion values reported here for flexion of the hip and ankle were similar to previously reported sagittal plane kinematics in healthy rats (Pereira et al., 2006). Pereira et al. reported average ranges of motion for flexion of the hip and ankle as roughly 45 and 50 degrees, which compare favorably to our observed values of 39.24 and 53.88 degrees.

Even with only eight rats, this method was sensitive enough to reveal significant differences in gait. The observed changes in gait pattern were similar to typical human gait compensation for foot drop in the forms of vaulting gait and circumduction. Vaulting gait is characterized by excessive hip and knee flexion, as well as pelvic hiking/obliquity. Some evidence of vaulting gait was observed at the 2- and 4-week timepoints as indicated by increased hip flexion during swing and ankle extension at toe-off. At the later timepoints, a compensatory shift to circumduction was observed. Circumduction is another compensation mechanism for foot drop that is more efficient than vaulting and is characterized by swinging a straight leg around the long axis of the body. This gait pattern was seen at the 8-week timepoint, as evidenced by increased hip abduction and external rotation as well as increased knee and ankle extension at toe-off and during swing.

Though we limited the motion of the knee to the sagittal plane, we do not believe that there would be statistically significant changes in knee varus/valgus in these animals due to a TA VML injury. Further, the range of motion of knee valgus in humans is <5 degrees(Cherian et al., 2014), which is less than the resolution in this study.

As indicated by the force testing results (Figure 4), the animals were operating at a significant functional deficit at all three post-surgical timepoints. This supports the rightward shift (i.e., more time in stance) in the gait cycle as the weeks progress, as the animal was forced to take more time to develop an impulse to drive the foot forward. The torque values in this study were slightly higher than those for NR animals in previous work (Passipieri et al., 2017), indicating a less severe VML injury but highlighting the sensitivity of the gait analysis presented here. The spatiotemporal results also confirm this functional deficit by showing a significant decrease in swing percentage of the gait cycle. Similarly, analysis of the spatiotemporal parameters showed that cadence increased even though the treadmill velocity remained the same, indicating the animals were trying to increase their stability as compensation for the injury. The gait analysis methods in this study were sensitive enough to show that compensation patterns were fluid throughout the 8 week observation period. The animals were utilizing circumduction as a compensation mechanism at the 8-week time point, however their gait patterns had not yet reached a steady state. While we believe the observed circumduction reflects the final learned or permanent gait pattern resulting in the most efficient mechanism of movement for the given VML injury, we are not able to make such a conclusion. Future studies should employ increased group sizes to improve statistical power and follow the evolution of the rat gait patterns to 16 or 24 weeks to confirm the gait patterns seen at 8 weeks were durable. Extending the final data collection timepoints out further and expanding the group sizes would likely reveal more differences and could provide beneficial information in quantifying the time it takes to reach a kinematic plateau.

This data is a promising start, but it could be improved in a few ways. We believe that using these same motion capture techniques on animals walking over-ground rather than on a treadmill would result in joint kinematics that are more reflective of natural gait patterns. While the shock was a necessary condition to motivate the animals and keep them on the belt, it also created a panic in the animals and led to markedly different walking styles for a brief period afterwards. In addition, the measurement of ground reaction forces via an instrumented walkway would allow us to calculate joint moments in all three planes. The inclusion of force plates, along with the collection of contralateral limb kinematics, would provide a more insightful data set and develop data which would give us an opportunity to evaluate the efficacy of various rehabilitative and regenerative strategies on a deeper functional level.

In spite of the small group sizes of this pilot study, the developed method for the analysis of rat locomotion resulted in a small variance between unique rats and unique visits for healthy controls and a high sensitivity to small differences between healthy and VML rats, demonstrating its value as an evaluative tool. We were able to accurately calculate the kinematics of the hip, knee, and ankle over complete gait cycles and compare the post-injury kinematics to healthy baseline data. This evaluation method allowed us to detect significant small differences in stance and swing at all three joints throughout the data collection period due to VML in the injured population. Based on these results, we can conclude that the differences observed after injury can be attributed to vaulting gait and circumduction as compensation for drop foot. We believe our method of quantifying functional ability should be considered ahead of the current industry standard of *in vivo* force testing for a variety of reasons, most notably that it quantifies functional recovery on a basis of movement function rather than just force generation ability. By allowing us to determine the variance of kinematic variables and the limitations of a treadmill-based motion capture arena, this study has established a groundwork for future studies into protocol refinement, joint kinetics, and comparisons of efficacy of regenerative therapeutics as treatment for VML and more significant injuries.

## Data Availability

All datasets generated for this study are included in the manuscript and/or the supplementary files.

## Ethics Statement

This study was conducted in compliance with the Animal Welfare Act, the Implementing Animal Welfare Regulations, and in accordance with the principles of the Guide for the Care and Use of Laboratory Animals. The University of Virginia Animal Care and Use Committee approved all animal procedures. A total of 8 female Lewis rats (Charles River Laboratories) weighing 180.2 ± 6.75 g at 12 weeks of age were pair housed in a vivarium accredited by the American Association for the Accreditation of Laboratory Animal Care, and they were provided with food and water *ad libitum*.

## Author Contributions

JD collected, analyzed, and interpreted the majority of the data in the study and wrote the manuscript. XH worked to modify the OpenSim hindlimb model that allowed for data analysis and provided approval for publication of the content. KJ contributed to a significant amount of data acquisition for the work. CS contributed to data acquisition and a significant amount of data analysis for the work. ED wrote code to extract spatiotemporal parameters from the gait trials and assisted in data acquisition for the work. GC and SR oversaw the conception, design, and funding of the work and also interpreted the results of the study and provided approval for publication of the content.

### Conflict of Interest Statement

The authors declare that the research was conducted in the absence of any commercial or financial relationships that could be construed as a potential conflict of interest.
